# Management of the infertile couple: an evidence-based protocol

**DOI:** 10.1186/1477-7827-8-21

**Published:** 2010-03-06

**Authors:** Remah M Kamel

**Affiliations:** 1 Department of Obstetrics and Gynaecology, Faculty of Medicine, Jazan University, Jazan, Saudi Arabia

## Abstract

**Background:**

Infertility is defined as inability of a couple to conceive naturally after one year of regular unprotected sexual intercourse. It remains a major clinical and social problem, affecting perhaps one couple in six. Evaluation usually starts after 12 months; however it may be indicated earlier. The most common causes of infertility are: male factor such as sperm abnormalities, female factor such as ovulation dysfunction and tubal pathology, combined male and female factors and unexplained infertility.

**Objectives:**

The aim of this study is to provide the healthcare professionals an evidence-based management protocol for infertile couples away from medical information overload.

**Methods:**

A comprehensive review where the literature was searched for "Management of infertility and/or infertile couples" at library website of University of Bristol (MetaLib) by using a cross-search of different medical databases besides the relevant printed medical journals and periodicals. Guidelines and recommendations were retrieved from the best evidence reviews such as that from the American College of Obstetricians and Gynaecologists (ACOG), American Society for Reproductive Medicine (ASRM), Canadian Fertility and Andrology Society (CFAS), and Royal College of Obstetricians and Gynaecologists (RCOG).

**Results:**

A simple guide for the clinicians to manage the infertile couples.

**Conclusions:**

The study deploys a new strategy to translate the research findings and evidence-base recommendations into a simplified focused guide to be applied on routine daily practice. It is an approach to disseminate the recommended medical care for infertile couple to the practicing clinicians.

## Background

Infertility is a common clinical problem. It affects 13% to 15% of couples worldwide [1]. The prevalence varies widely, being less in developed countries and more in developing countries where limited resources for investigation and treatment are available [2]. In the United Kingdom, it is estimated that one in six couples would complaint of infertility [3].

In addition, infertility is considered also a public problem. It does not affect the couples' life only, but it also affects the healthcare services and social environment [4]. The feelings experienced by the infertile couples include depression, grief, guilt, shame, and inadequacy with social isolation.

Today, many patients do not receive the recommended medical care that based on the best available evidence [5]. As there is surplus medical information everyday that can not be cached by healthcare providers, clinical guidelines created to endorse *up-to-date *evidence-based practice to improve patients' outcome [6].

This study was carried out in accordance with the requirements of the University of Bristol Regulations and Code of Ethics for Research Programmes.

### Methodology

This paper, as a comprehensive review, deploys a new strategy to translate the research findings and evidence-base recommendations into a simplified focused guide to be applied on routine daily practice. It is an approach to disseminate the recommended medical care of infertile couple to the practicing clinicians. To accomplish this, the literature was searched for the keywords of "*Management of infertility, infertile couples*" at library website of University of Bristol (MetaLib) by using a cross-search of different medical databases such as ...Allied and Complementary Medicine Database (AMED), ...BIOSIS Previews on Web of Knowledge, ...Cochrane Library, ...PubMed, including Medline, and ...Web of Science, in-addition to the relevant printed medical journals and periodicals. Guidelines and recommendations were retrieved from the best evidence reviews at the American College of Obstetricians and Gynaecologists (ACOG), American Society for Reproductive Medicine (ASRM), Canadian Fertility and Andrology Society (CFAS), European Society of Human Reproduction and Embryology (ESHRE), Human Fertilisation and Embryology Authority (HFEA), Royal College of Obstetricians and Gynaecologists (RCOG), and the World Health Organization (WHO).

### Epidemiology of infertility

For healthy young couples, the probability of getting pregnancy per a reproductive cycle is about 20% to 25%. Their cumulative probabilities of conception are 60% within the first 6 months, 84% within the first year, and 92% within the second year of regular fertility-focused sexual activity.

Several studies have reported different causes of infertility [7-9]. Some causes are more common in some countries than others, such as pelvic inflammatory diseases (PID) and sexually transmitted infections (STI) in Africa [10]. Some personal habits are considered risk factors for infertility, such as excess alcohol intake [11] and cigarette smoking [12].

According to the literature survey, the most common causes of infertility are: male factor [5,7-9,13-15] such as sperm abnormalities [9,13,15], female factor [7-9,14-16] such as ovulation dysfunction [7,8] and tubal pathology [7-9], combined male and female factors [7,9,14,15] and unexplained infertility; where no obvious cause could be detected [7-9].

As the rate of getting spontaneous pregnancy among infertile or subfertile couples is lower than that among normal fertile population, it is recommended to carry out the following diagnostic, *evidence-based*, work-up to detect any hidden treatable cause.

### History-taking

Couples with infertility problem should be interviewed separately as well as together, to bring out important facts that one partner might not wish to disclose to the other. Full history taking of both partners usually denotes the underlying problem [17-23], (Appendix 1).

### Clinical examination

Full clinical examination of both partners usually stands for the underlying physical problem [17-22,24-26], (Appendix 2). By the end of this step, most of healthcare professionals will be able to sketch out their provisional diagnosis. Investigations will be requested to prove the clinical diagnosis and to exclude other close possibilities.

### Investigations

Infertile couples are usually adviced to start their investigations after 12 months of trying to conceive or after 6 months if the female partner is more than 35 years old or immediately if there is an obvious cause for their infertility or subfertility [16].

As the major causes of infertility are sperm abnormalities, ovulation dysfunction, and fallopian tube obstruction, the preliminary adviced investigations for the infertile couple should be focused on semen analysis (to be compared with the WHO reference values [27]), detection of ovarian function by hormonal assay (early follicular FSH and LH levels, and mid-luteal progesterone), and evaluation of tubal patency by hysterosalpingography (HSG) [17-32], (Appendix 3).

Many infertile couples have had some previous assessment for their infertility and this data should be cautiously reviewed. Further investigations may be requested according to the clinical presentation and the results of preliminary tests. Omitting unnecessary investigations, in particular couples, could reduce total cost of their infertility management without compromising their success rate. For example; a woman who has no history suggestive of previous pelvic inflammatory disease or endometriosis, there is no justification to request a laparoscopy especially after normal hysterosalpingography study [33]. Similarly, there is no need for testing tubal patency for couples who will require IVF or ICSI procedure.

A woman with a suspicion of chronic anovulation most probably due to polycystic ovary (PCO) syndrome, as there is a long history of irregular cycles and clinical presentation with hirsutism, her serum levels of testosterone hormone, sex hormone binding globulin (SHBG), dihydroepiandrostenedione (DHEA), dihydroepiandrostenedione-sulfate (DHEAS) and prolactin should be evaluated to prove the provisional diagnosis and to detect the source of excess androgens. However, early referral of infertile couples to a dedicated specialist infertility clinic may be indicated to increase their chance of pregnancy (Table 1).

**Table 1:** Criteria for early referral to specialist infertility clinic

In women	In men
Age: < 35 years with > 18 months infertility.	History of: Genital pathology
≥35 years with > 6 months infertility	Uro-genital surgery
	Sexually-transmitted infections
	Varicocele
	Cryptorchidism
Length of menstrual cycle: < 21 days.	Systemic illness
> 35 days	Chemotherapy/Radiotherapy
Menstrual abnormalities: Amenorrhoea	
Oligomenorrhoea	
History of: Ectopic pregnancy	Two abnormal results of semen analysis:
Pelvic infections (PID)	Sperm count < 20 million/ml
Endometriosis	Sperm motility < 25% (grade-a)
Pelvic surgery (ruptured appendix)	Sperm motility < 50% (grade-b)
Developmental anomalies	Sperm morphology < 15% normal
Abnormal P/V findings on examination	Abnormal findings on genital examination
Chlamydia antibody titre ≥ 1:256	Patient request or Anxiety
Mid-luteal progesterone < 20 nmol/l	
FSH > 10 IU/l early follicular phase	
LH > 10 IU/l early follicular phase	
Patient request or Anxiety	

In some cases, the cause of infertility or subfertility could not be suspected from the history taking and clinical examination. In such circumstances, it is recommended not to prescribe any medication until all basic investigations are done and its results received.

### Treatment

The cost of infertility treatment is high [34]. For this reason, a call for low-cost ART protocols have been attempt to reduce the overall current cost of IVF through limiting the rquired laboratory investigations, modifying the stimulation regimen and purchasing low-priced pre-used machines and instruments [35]. But the question that should be answered one day is: will the output quality be compromised with such approach?

With the fast progression in reproductive medicine and the experiences gained through infertility management, a wider range of treatment options have become available to infertile couples [17-19,21-26,31,36], (Appendix 4). There are three main types of fertility treatment: medical treatment (such as ovulation induction therapy); surgical treatment (such as laparoscopy and hysteroscopy); and the different assisted reproduction techniques [37].

Choice of infertility treatment often related to issues of efficacy, cost, ease of use or administration, and its side effects. Legal, cultural and religious inquiries have limited the available choices in some countries, such as the use of donor sperms or oocytes.

Treatment options available for any particular infertile couple will depend also on the duration of their infertility, which partner is affected, the age of the female partner and if any has a previous children or not, the underlying pathological cause, and if the treatment will be covered by the National Health System (NHS) or funded by their own.

### Counseling

Fertility clinics should address the psycho-social and emotional needs of infertile couples as well as their medical needs. The content of counselling may differ depending on the concerned couple and the existing treatment options. It usually involves treatment implication counselling, emotional support counselling, and therapeutic counselling [38,39].

Most of infertile couples are aware of what can be offered to them from the media. Regrettably, this often leads to untruly high expectations of assisted reproduction techniques (ART) [40]. The chance of a live birth following treatment is nearly 50% [25]. It varies with the age (the optimal female age is between 23 and 39 years) and with body weight (the ideal body mass index is between 19 and 30). It is more successful in women who have previously been pregnant. There is no reliable means to predict whether the use of any treatment option will be successful and after how many attempts. The facilities available and the skills of personnel are the major determining factors for the success rate.

An estimated 28% of all couples seeking reproductive assistance may have normal findings on their clinical evaluation, making the unexplained infertility a more common provisional diagnosis. The predictable pregnancy rate for this group is about 5% after timed intercourse, 10% after superovulation with intrauterine insemination (IUI), and 15% to 25% after assisted reproduction techniques (ART) [41]. These rates, of course, readjusted down for the older women with long durations of infertility [42].

As treatment begins, couples may experience cycles of optimism and despair with each passing menstrual cycle. As the duration of treatment prolonged, psychological suffering is likely to increase [6]. The treating doctor may feel inadequacy and the trust between the doctor and patient breaks down [43]. At this point, psychological consultation and support should be provided [44].

The incidence of congenital malformation in IVF babies ranges between 2% and 3% worldwide and is similar to that in babies conceived naturally [45]. However there is a minimal increased risk of *de-novo *chromosomal abnormalities in ICSI born babies [46,47] that necessitate counselling of the concerned couples.

## Conclusions

Infertility by itself does not threaten the life, but it has devastating psycho-social consequences on infertile couples. It remains a worldwide problem challenge. Management of infertility has been and still a difficult medical task not only because of the difficulty in the diagnosis and treatment of the reproductive disorders in each partner, or the poorly unstated interaction between the partners' fertility potentials, but also because of the fact that success of treatment is clearly identifiable entity; the achievement of pregnancy. The treating doctor who is counselling the couple regarding their infertility must be familiar with the causes, investigations and the treatment options available. The couple needs to be given realistic information about their chances of having a live birth, as well as, the risks and costs of the management plan and its alternatives. By follow the proposed, *evidence-based*, management protocol stated in this paper, infertile couples will have a good chance to start up their treatment in the proper way at early time with enough financial support through reducing money spent on unnecessay investigations.

## Competing interests

The authors declare that they have no competing interests.

## Authors' contributions

This work was done by RMK and there is no contribution of any other authors.

## Appendix 1. Focused history taking for infertile couple

### **Female and Male Partners**

Female Partner

**Present history**: the current problem/complaint, age, occupation, recent cervical smear findings, breast changes as milk-like discharges, excessive hair growth with or without acne on face and chest, hot flushes, eating disorders, any current associated medical illness as diabetes and/or hypertension, drug intake prescribed as non-steroidal anti-inflammatory drugs (NSAIDs), sex steroids and cytotoxic drugs or recreational as marijuana and cocaine, smoking, alcohol, and caffeine consumption

**Menstrual history**: for age of menarche, cycle characteristics and any associated symptoms as painful menstruation or intermenstrual spotting. History of primary or secondary amenorrhoea

**Obstetric history**: previous pregnancies, *if any*, and its outcome, recurrent pregnancy loss, induced abortion, post-abortive infection or puerperal sepsis

**Contraceptive history**: previous use of any contraceptive method, particularly intrauterine system, and any associated problems

**Sexual history**: Coital frequency, timing in relation to the cycle, use of vaginal lubricant before, or vaginal douching after, coitus, loss of libido, as well as, any associated problems as difficult or painful coitus

**Past history**: medical or surgical as pelvic infection, tuberculosis, bilharziasis, ovarian cyst, appendicectomy, laparotomy, caesarean sections, and cervical conization. Ask about her rubella status

**Family history**: for similar problem among the female members, consanguinity, diabetes mellitus, hypertension, twins delivery, breast cancer

Male Partner

**Present history**: the current problem/complaint, age, occupation, previous seminal analysis findings, breast changes as enlargement, any current associated medical illness as diabetes and/or hypertension, drug intake prescribed or recreational, smoking, alcohol, and caffeine consumption

**Sexual history**: Coital frequency, timing, and any associated problems as erectile dysfunction or ejaculatory problems, loss of libido. History of previous marriage or extra-marital sexual relations

**Contraceptive history**: previous use of any contraceptive method either temporary as condom or permanent as vasectomy

**Past history**: medical disease or surgical operations as mumps, tuberculosis, bilharziasis, sexually-transmitted infections, hydrocele, varicocele, undescended testis, appendicectomy, inguinal hernia repair, or bladder-neck suspension operations

**Family history**: for similar problem among the male members, consanguinity, diabetes mellitus, and hypertension

## Appendix 2. Focused clinical examination for infertile couple

### **Female and Male Partners**

Female Partner

**General Examination**: vital signs (especially blood pressure), body height and weight (BMI= ratio between weight in kilograms and height in square meters) for over or under weights, secondary sexual characters, any excessive hairs with/without acne on face or chest, and acanthosis nigricans. Abnormal skin depigmentation as vitiligo may suggest an autoimmune systemic disease. Examination should include also the thyroid gland

**Breast Examination**: to evaluate its development and to exclude any pathology or presence of occult galactorrhoea

**Chest Examination**: for lungs and heart

**Abdominal Examination**: for any abdominal mass, organomegaly, ascites, abdominal striae, and surgical scars

**Genital Examination**: type of circumcision, size and shape of clitoris, hymen, vaginal introitus, site, size, shape, surface, consistency, mobility and direction of uterus, any palpable adnexal mass, vaginal discharge, tenderness, uterosacral ligament thickening, and nodules in the cul-de-sac denoting either endometriosis or tuberculosis by per-vaginal (PV) examination

Male Partner

**General Examination**: vital signs (especially blood pressure), body height and weight (BMI), arm-span, secondary sexual characters, and examination of thyroid gland.

**Breast Examination**: for gyanecomastia

**Abdominal Examination**: for any abdominal mass, undescended testis, inguinal hernia, organomegaly, or ascites

**Genital Examination**: shape and size of penis, prepuce, position of external urethral meatus, testicular volume (by using Prader's Orchidometer. Normally 25 ml = 3 × 5 cm), palpation of epididymis and vas deferens, exclude varicocele or hydrocele. Perineal sensation, rectal sphincter's tone, and prostate enlargement by per-rectal (PR) examination

## Appendix 3. Focused investigations for infertile couple

### **Female and Male Partners**

Female Partner

Basic Investigations

**General**: Full blood count, urine analysis, Papanicolaou smear, vaginal wet mount with appropriate culture, Rubella serology, Hepatitis B and C, HIV serology, and *Chlamydia trachomatis *serology

**Hormonal assay**: to predict ovulation and ovarian reserve. Mid-luteal serum progesterone level (5-10 days before the expected menstrual cycle). FSH, LH (twice if female age > 38 years, on day 2-5 of the menstrual cycle). The use of basal body temperature (BBT) charting and ovulation predictor home kits are not recommended

**Transvaginal ultrasonography**: to monitor natural ovulation, to detect any pelvic pathology as uterine or ovarian masses, abnormally-shaped or mal-directed uterus. No need for ultrasound scanning of endometrium

**Hysterosalpingography **or **Hysterosalpingo-Contrast-Sonography (HyCoSy)**: to evaluate shape of uterine cavity and patency of both fallopian tubes in low-risk women

Advanced Investigations

**Hormonal assay**: Prolactin (if cycles are irregular with/without galactorrhoea or pituitary adenomas). Thyroid function tests (for women with symptoms of thyroid disease). Testosterone, SHBG, DHEA and DHEAS (for suspected cases with PCO syndrome)

**Laparoscopy**: for possible associated pelvic pathology or adhesions in cases with abnormal HSG findings, previous history of pelvic inflammatory disease or endometriosis

**Hysteroscopy**: for intrauterine space-occupying lesions detected on HSG as adhesions or polyp (no evidence linking it with enhanced fertility)

**Chromosomal karyotyping**: for suspected genetic disorders as Turner's syndrome

Male Partner

Basic Investigations

**General**: Full blood count, Hepatitis B and C, HIV serology, and *Chlamydia trachomatis *serology

**Semen analysis **(after 72 hours of sexual abstinence): interpreted for its volume, sperm count, motility, and morphology according to the WHO reference values (Two analyses with 3 months apart at the same lab)

Advanced Investigations

**Post-coital test**: no predictive value on the pregnancy rate

**Anti-sperm antibodies **(no evidence of effective treatment to improve fertility), and **Sperm function tests**

**Hormonal assay**: FSH, LH, Testosterone, TSH and Prolactin (for male with abnormal seminal analysis and suspected endocrine disorder)

**Testicular biopsy**: A fine-needle aspiration biopsy may required to differentiate between obstructive and non-obstructive azoospermia

**Chromosomal karyotyping**: for suspected genetic disorders as sex chromosomal aneuploidy, cystic fibrosis, and deletion of Y-chromosome

## Appendix 4. Treatment Options for Infertile couple

### **Female and Male Partners**

Female Partner

Non-Invasive Treatment

**Counselling**: for regular intercourse 2-3 times/week. Give-up smoking, not to drink more than 1-2 units of alcohol/week, not to use any addictive drugs, and follow a supervised weight loss programme if obese (BMI > 29). Folic acid 0.4 mg should be provide as a daily supplement to prevent neural tube defect (5.0 mg adviced for women who have previously affected child or on medication for epilepsy). Rubella vaccination if seronegative (avoid pregnancy for one month). Treat any psycho-sexual problem if present

**Induction of Ovulation**: for women with ovulatory dysfunctions. Provide a controlled ovarian stimulation for assisted reproduction techniques

**Intra-uterine Insemination (IUI)**: is not regulated by Human Fertilisation and Embryology Authority (HFEA) in the United Kingdom. Could be used for unexplained infertility and female cases with minimal endometriosis

**Surrogacy**: In women with congenital absence of uterus or after surgical removal

Invasive Treatment

**Tubal surgery**: as laparoscopic adhesiolysis, tubal cannulation or catheterisation

**Hysteroscopic surgery**: as resection of IU adhesions or polyp

**In-vitro Fertilisation (IVF) **and **Embryo transfer (ET)**: Used procedure for female tubal factor, moderate male factor, and for unexplained infertility

**Gamete Intra-fallopian Transfer (GIFT) **and **Zygote Intra-fallopian Transfer (ZIFT)**: are not regulated by HFEA. For cases who refuse fertilisation in lab

**Oocyte donation **and **Ovarian tissue transplantation**: for premature ovarian failure

Male Partner

Non-Invasive Treatment

**Counselling**: for regular intercourse 2-3 times/week, give-up smoking, reduce alcohol intake to 3-4 units/week, not to use any addictive drugs, wear loose fitting underwear and trousers, and avoid occupational or social situations that might cause testicular heating. Treat any psycho-sexual problem if present

**Intra-uterine Insemination (IUI)**: Used for mild male factor infertility problems

**Donor insemination**: for azoospermia, positive HIV, severe male factor who refuse the ICSI as a management option

**Adoption**: For cases with recurrent unexplained failed IVF cycles

Invasive Treatment

**Surgical restoration of duct patency**: For cases with previous vasectomy

**Intra-cytoplasmic Sperm Injection (ICSI) ± PGD**: Commonly used procedure for severe male factor or for recurrent unexplained failed IVF cycles. Surgical sperm retrieval (SSR) usually done by percutaneous epididymal sperm aspiration (PESA), testicular sperm aspiration (TESA), testicular sperm extraction (TESE), or microsurgical epididymal sperm aspiration (MESA).
